# State Estimation of the Time-Varying and Spatially Localized Concentration of Signal Molecules from the Stochastic Adsorption Dynamics on the Carbon Nanotube-Based Sensors and Its Application to Tumor Cell Detection

**DOI:** 10.1371/journal.pone.0141930

**Published:** 2015-11-03

**Authors:** Hong Jang, Jay H. Lee, Richard D. Braatz

**Affiliations:** 1 Department of Chemical and Biomolecular Engineering, Korea Advanced Institute of Science and Technology, Daejeon, Korea; 2 Department of Chemical Engineering, Massachusetts Institute of Technology, Cambridge, Massachusetts, United States of America; Wake Forest University School of Medicine, UNITED STATES

## Abstract

This paper addresses a problem of estimating time-varying, local concentrations of signal molecules with a carbon-nanotube (CNT)-based sensor array system, which sends signals triggered by monomolecular adsorption/desorption events of proximate molecules on the surfaces of the sensors. Such sensors work on nano-scale phenomena and show inherently stochastic non-Gaussian behavior, which is best represented by the chemical master equation (CME) describing the time evolution of the probabilities for all the possible number of adsorbed molecules. In the CME, the adsorption rate on each sensor is linearly proportional to the local concentration in the bulk phase. State estimators are proposed for these types of sensors that fully address their stochastic nature. For CNT-based sensors motivated by tumor cell detection, the particle filter, which is nonparametric and can handle non-Gaussian distributions, is compared to a Kalman filter that approximates the underlying distributions by Gaussians. In addition, the second-order generalized pseudo Bayesian estimation (GPB2) algorithm and the Markov chain Monte Carlo (MCMC) algorithm are incorporated into KF and PF respectively, for detecting latent drift in the concentration affected by different states of a cell.

## Introduction

Recently, several near-infrared (nIR) fluorescent sensors based on single-walled carbon nanotubes (SWNTs) have been developed for detecting biomolecules in the human body [1–9]. In response to a continuous incident light source, the SWNT-based sensors detect stepwise changes in emitted light intensity triggered by monomolecular adsorption and desorption (i.e., adsorption and desorption at a single-molecular level) of a trace of proximate molecules on the surfaces of the sensors. The nIR fluorescence can penetrate more deeply into tissues than visible fluorescence without photobleaching or overlapping with autofluorescence from biological substrates [10, 11]. Furthermore, compared with small fluorescent probes [12–22], non-diffusive SWNTs allow for a precise spatial resolution at the micrometer scale. As a result of these advantages, SWNTs can act as effective sensing platforms for real-time, direct and selective detection *in vivo*. In particular, for nitric oxide (NO) and hydrogen peroxide (H_2_O_2_), μM level concentration could be detected successfully by using this sensing platform and resolve several questions about local generation upon growth factor stimulation and the signalling mechanism in a living cell [7, 8].

This sensor technology presents some challenges as well as opportunities. A sensor array system where multiple CNT-based sensors distributed on a small area potentially can be used to follow time-varying, local concentrations of target molecules *in vivo* and in real time with precise spatial resolution. In turn, precise spatiotemporal control of these molecules may become feasible with the advent of appropriate actuators. Challenges in the experimental side include selective sensor design for target molecules in a desired detection range and actuator design for the spatiotemporal control at micro-scale. On the system’s side, an immediate challenge is the development of an on-line state estimation method that can effectively extract concentration information from the stochastic adsorption data.

Some methods have been proposed for quantifying local concentrations of signal molecules near CNT-based sensors [23–25]. The estimation task is made challenging by the fact that the adsorption/desorption event is highly stochastic given a small number of molecules involved at the nanoscale sensor’s surface. Conventional methods like least squares are limited in terms of performance for such problems. For a more accurate estimation, chemical master equation (CME) describing the evolution of the probability distribution among all possible adsorption states (i.e., the number of adsorbed molecules on the sensor) has been used in the estimation formulation. Based on the exact solution of the CME, maximum likelihood estimation (MLE) has been proposed [23–25]. However, the previous works assumed a constant concentration and performed the estimation with a batch set of data, which is not realistic for a sensor system working in a real-time environment in which concentrations show dynamic, time-varying behavior. What is needed is a full state estimation method that can fully and recursively utilize the information coming from the sensors to follow the local concentration in real time.

Bayesian methods have been a popular choice for state estimation of stochastic systems owing to its flexible, convenient formulation and theoretical rigor. For Gaussian systems, only the first two moments of the probability density function (PDF) have to be followed and the Kalman filter (KF) provides a simple solution to the problem. However, data from the CNT-based sensor system shows highly non-Gaussian characteristics that follow convolved binomial distributions [24]. For highly non-Gaussian systems, a class of sequential Monte Carlo methods known as particle filters (PFs) can be attractive as a nonparametric method that can handle any distribution shape [26]. The PF methods represent the required posterior PDF as a set of random samples and associated weights.

This article mainly proposes an effective recursive state estimator for estimating time-varying, local concentrations of signal molecules using the stochastic adsorption and desorption time-profiles onto the surface of the CNT-based sensors. By tracking the concentration of the signal molecules with the help of a rigorously formulated stochastic state estimator, we can gain further insights into their roles in biological systems or the effects of other species on them. The stochastic nature of the adsorption and desorption at the molecular level brings in the chemical master equation (CME) at the sensor level and makes the problem a challenging one that cannot be easily handled by the conventional state estimation techniques. Hence, the state estimation problem studied in this article has not been addressed before in the literature.

To test the feasibility and potentials of the proposed method, we test it in the context of a sensing problem, which is admittedly simplistic and artificial but still is inspired by the real biological problem. Given the known parameters in the model, performances of the KF and PF methods are examined in terms of both accuracy of estimated local concentration of the signal molecule and computational cost. The nano-sensors have previously been used for detecting and measuring signal molecules in human body, to follow the concentrations of signal molecules like nitric oxide (NO) and hydrogen peroxide (H_2_O_2_), which are consistently generated from enzymes in vascular endothelial cells to regulate various physiological and pathological processes [6,23–25]. Their concentration levels are known to be affected significantly by cell states, the switching behavior of which is simplistically represented by a hidden Markov model in our case study. To solve the simulated estimation problem, KF and PF are designed with the second-order generalized pseudo-Bayesian estimation (GPB2) algorithm and the Markov chain Monte Carlo (MCMC) algorithm respectively, for the Markov jump system with nano-sensors. Their performances are compared for the case of a single sensor as well as of multiple sensors.

## Methods

### Single-molecule Sensor System

#### Carbon nanotube-based sensor

**Fig 1:**
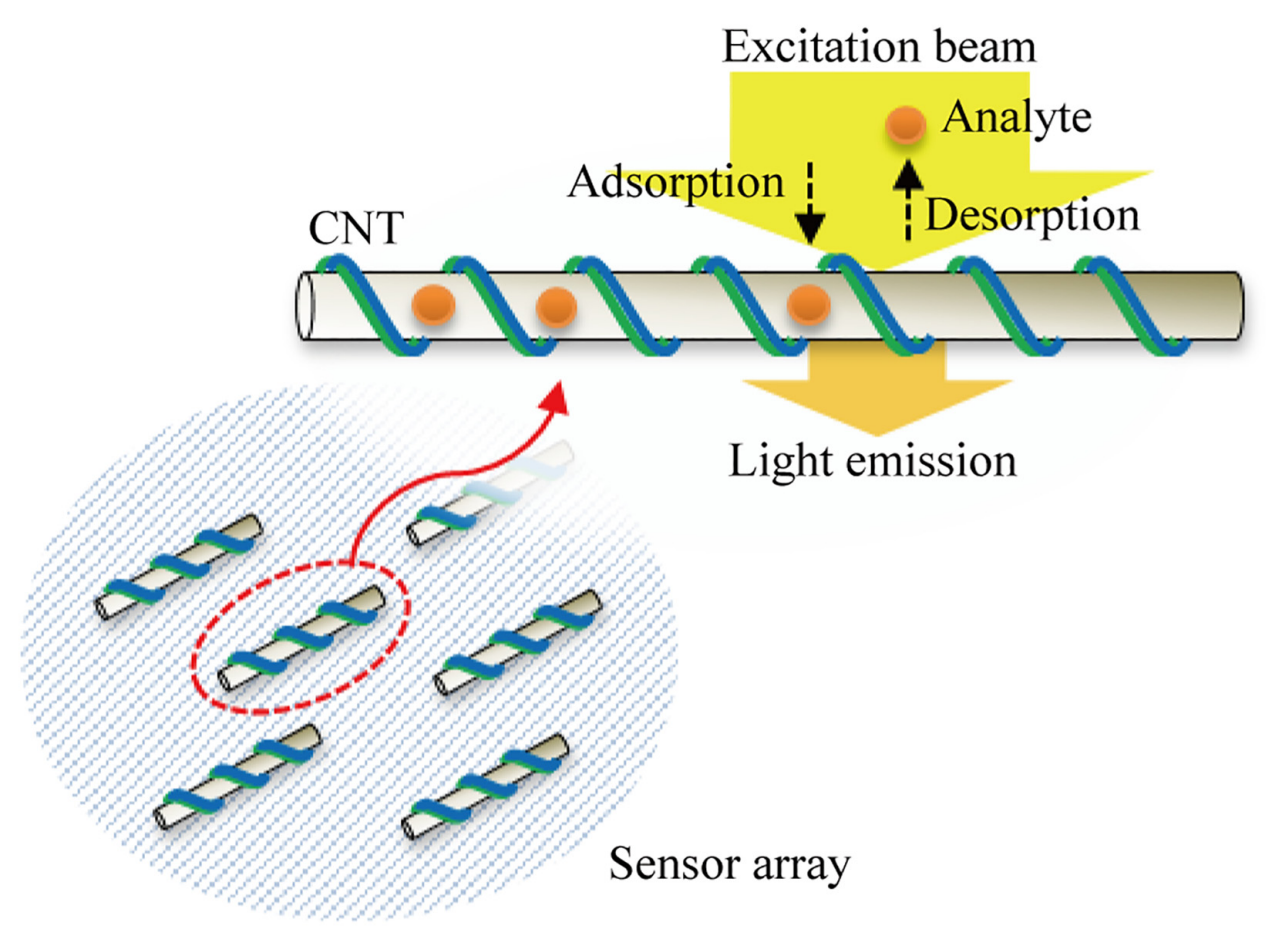
An example of a SWNT-based sensor array system.

The basic mechanism of SWNT-based sensors is optical detection of discretized light intensity changes induced by adsorption and desorption of target molecules on the sensor’s surface at nano-scale. To enhance the sensitivity and selectivity for target molecules, usually present at the micromolar (μM) concentration level, the SWNT surface is functionalized by wrapping the nanotube with various polymers such as collagen [7] or certain DNA sequences [8] (Fig 1). The variation in the SWNT wrapping controls the adsorption rates of different analytes present. For example, collagen-SWNTs have shown different, selective time-profiles of adsorption and desorption events for H_2_O_2_, H^+^, and Fe(CN)_6_^3−^ in different concentration ranges [6]. Importantly, all time-profile data had reversible features, which indicate adsorption and desorption rates of similar magnitudes.

The maximum number of adsorbed molecules is experimentally found to be around 10 [8], and this number is consistent with the maximum number of excitons (an excition is an electron and positive hole pair, which remain near each other due to electrostatic Coulomb force and is free to move through a semiconducting material) diffusion-limited segments on the SWNT [1] for which an average length is about 1~2 μm. So several SWNT-based sensors can be placed in a small area less than 10 μm^2^ [8]. Fig 1 shows an example of a sensor array system depicted as sensors randomly distributed on a small area of neighborhood. With this array system, the objective is to estimate a time profile of the local concentration of target molecules with high accuracy.

#### Stochastic adsorption model

The number of adsorbed molecules is assumed to be read at every sampling time from the sensors, which are distributed in a sufficiently small area of a same concentration level. In developing a sensor model, free target molecules *A* in its surrounding liquid phase are assumed to adsorb onto unoccupied sites of the nanotube segment *θ* to form bound molecules *Aθ* through reversible adsorption:

$$A+\theta \begin{array}{c} k_{A}^{'}\\ ⇌\\ k_{D} \end{array}A\theta$$ (1)

where $k_{A}^{'}$ [s^−1^] and *k*_*D*_ [s^−1^] are adsorption and desorption rate constants, respectively. The corresponding rates are expressed as

$$r_{A}=k_{A}^{'}N_{\theta }$$ (2)

$$r_{D}=k_{D}N_{A\theta }$$ (3)

where *N*_*θ*_ is the number of empty sites and *N*_*Aθ*_ is the number of occupied sites. The adsorption rate can be considered to be a first-order function of the local concentration of the surrounding target molecules *C*(*t*) [24],

$$k_{A}^{'}=k_{A}C\left(t\right)$$ (4)

where *k*_*A*_ is a constant factor in the adsorption coefficient.

These equations connect the sensor information (i.e., the number of absorbed molecules) to the concentration in the surrounding media. If the adsorption/desorption events could be deterministic, a continuum (or average) model for the sensor can be formed by one differential equation for the number of adsorbed molecules *N*_*Aθ*_ ∈ [0, *N*_*T*_] as a continuous variable with an initial value of *N*_*Aθ*,0_,

$$\frac{dN_{A\theta }\left(t\right)}{dt}=k_{A}C\left(t\right)\left(N_{T}-N_{A\theta }\left(t\right)\right)-k_{D}N_{A\theta }\left(t\right),\;N_{A\theta }\left(0\right)=N_{A\theta ,0}$$ (5)

A recursive form of the solution obtained by considering the previous measurement *N*_*Aθ*_(*t*_*k*−1_) as an initial condition and integrating the equation for one sample interval assuming *C*(*t*_*k*_) remains constant over the interval is

$$N_{A\theta }\left(t_{k}\right)=\frac{N_{T}+\left(N_{A\theta }\left(t_{k-1}\right)\left(1+\frac{k_{D}}{k_{A}C\left(t_{k}\right)}\right)-N_{T}\right)e^{-\left(k_{A}C\left(t_{k}\right)+k_{D}\right)\Delta t}}{1+\frac{k_{D}}{k_{A}C\left(t_{k}\right)}}$$ (6)

where *k* is the index for the time step and Δ*t* is the size of the sample time step size, which is set sufficiently small for the approximation to be accurate.

In actuality, the adsorption reaction on the sensor surface is highly stochastic because only a very small number of molecules (~10) are involved. Hence, significant fluctuations occur from the average behavior described in (6). In this case, use of the chemical master equation (CME) composed of differential equations describing the evolution of the probabilities for all possible discrete states of the system is more appropriate [27]. Then, the state of the system is defined as the discrete number of adsorbed molecules ${\tilde{N}}_{A\theta }\in \left[0,N_{T}\right]$, resulting in *N*_*T*_ +1 total possible states. The probability of being in each state is denoted by $P_{i}=\text{Pr}\left({\tilde{N}}_{A\theta }=i\right)\in \left[0,1\right]$, where *i* is the number of adsorbed molecules. The CME, along with the appropriate boundary equation, can be expressed by *N*_*T*_ +1 ordinary differential equations (ODEs):

$$\frac{dP_{0}\left(t\right)}{dt}=-P_{0}\left(t\right)\left[k_{A}^{'}N_{T}\right]+P_{1}\left(t\right)\left[k_{D}\right]$$ (7)

$$\frac{dP_{i}\left(t\right)}{dt}=P_{i-1}\left(t\right)\left[k_{A}^{'}\left(N_{T}-(i-1)\right)\right]-P_{i}\left(t\right)\left[k_{D}i+k_{A}^{'}\left(N_{T}-i\right)\right]+P_{i+1}\left(t\right)\left[k_{D}\left(i+1\right)\right],\;i=1,2,\ldots ,N_{T}-1$$ (8)

$$\frac{dP_{N_{T}}\left(t\right)}{dt}=P_{N_{T}-1}\left(t\right)\left[k_{A}^{'}\right]-P_{N_{T}}\left(t\right)\left[k_{D}N_{T}\right]$$ (9)

The monomolecular reaction systems, which were studied by [28], provide a path to an analytical solution of the CME. The adsorption/desorption process can be considered as a monomolecular reaction system with only two species (e.g. adsorbed molecules on sensor surface and desorbed molecules in bulk). For such a system, the probability distribution of the CME is described by a binomial distribution with time-varying parameters. More specifically, the number of adsorbed molecules *N*_*Aθ*_ at a time *t*_*k*_ is a random variable distributed as a binomial with the number of trials equal to *N*_*T*_ and probability parameter equal to *λ*(*t*_*k*_), which is related to *N*_*Aθ*_(*t*_*k*_) calculated from the continuum Eq (6) divided by *N*_*T*_ as

$${\tilde{N}}_{A\theta }\left(t_{k}\right)∼B\left(N_{T},\lambda (t_{k})\right)$$ (10)

$$\text{Pr}\left({\tilde{N}}_{A\theta }\left(t_{k}\right)=i\right)=\left(\begin{array}{c} N_{T}\\ i \end{array}\right){\left(\lambda (t_{k})\right)}^{i}{\left(1-\lambda (t_{k})\right)}^{N_{T}-i}$$ (11)

$$\lambda \left(t_{k}\right)=\frac{N_{A\theta }\left(t_{k}\right)}{N_{T}}$$ (12)

The local concentration of target molecule *C*(*t*_*k*_) enters the probability distribution of Eq (10) through *N*_*Aθ*_(*t*_*k*_) of Eq (6) appearing in Eq (12) for *λ*(*t*_*k*_).

For monomolecular adsorption, the overall population can be divided into two subsets, representing occupied sites and unoccupied sites on the sensor. With some previously measured value ${\hat{N}}_{A\theta }\left(t_{k-1}\right)$, the distribution at the next time step can be derived as the convolution of two binomial distributions applicable to the “fully occupied” and “empty” subsets, which are of size ${\hat{N}}_{A\theta }\left(t_{k-1}\right)$ and $1-{\hat{N}}_{A\theta }\left(t_{k-1}\right)$ respectively:

$${\tilde{N}}_{A\theta }(t_{k})∼B\left({\hat{N}}_{A\theta }(t_{k-1}),\lambda^{F}(t_{k})\right)*B\left(N_{T}-{\hat{N}}_{A\theta }(t_{k-1}),\lambda^{E}(t_{k})\right)$$ (13)

$$\lambda^{F}\left(t_{k}\right)=\frac{1+\frac{k_{D}}{k_{A}C\left(t_{k}\right)}{\text{e}}^{-\left(k_{A}C\left(t_{k}\right)+k_{D}\right)\Delta t}}{1+\frac{k_{D}}{k_{A}C\left(t_{k}\right)}}$$ (14)

$$\lambda^{E}\left(t_{k}\right)=\frac{1-{\text{e}}^{-\left(k_{A}C\left(t_{k}\right)+k_{D}\right)\Delta t}}{1+\frac{k_{D}}{k_{A}C\left(t_{k}\right)}}$$ (15)

The first binomial distribution can be derived from (10)–(12) by assuming the sites are fully occupied initially, and the second binomial distribution can be derived from (10)–(12) by considering the initial state as being empty [28]. If the expression for *N*_*Aθ*_(*t*_*k*_) obtained by setting *N*_*Aθ*_(*t*_*k*−1_) = *N*_*T*_ (“fully occupied”) in (6) is further substituted into (12), the probability parameter *λ*(*t*_*k*_) becomes *λ* ^*F*^(*t*_*k*_) of (14) and *N*_*T*_ cancels out. If the same substitution is carried out by setting *N*_*Aθ*_(*t*_*k*−1_) = 0 (“fully empty”) in (6), *λ*(*t*_*k*_) becomes *λ* ^*E*^(*t*_*k*_) of (15).

### Recursive State Estimation Design

Based on the observation model proposed in Section 2, the overall system for state estimation can be generally described by the discrete-time state space model,

$$x_{k}=f\left(x_{k-1}\right)+w_{k}$$ (16)

$$y_{k,j}∼p\left(y_{k,j}|x_{k},y_{k-1,j}\right),\;\forall j=1,\ldots ,N_{s}$$ (17)

where *x*_*k*_ is a single state indicating the local concentration *C*(*t*_*k*_) in the neighborhood; *w*_*k*_ is zero-mean white noise; *y*_*k*,*j*_ is the measurement of the number of adsorbed molecules ${\tilde{N}}_{A\theta ,j}\;\left(t_{k}\right)$ onto the surface of the *j*th sensor; *N*_*s*_ is the number of sensors in the neighbourhood, *f*(⋅) represents the state transition function which can describe production, degradation, mass transport, biological reactions, etc. of the signal molecules; and *p*(⋅) denotes the probability distribution represented by the convolution of the two binomial distributions, as in (13), which describes the stochastic adsorption reaction model. The expression involves both *x*_*k*_ and *y*_*k*−1,*j*_ (corresponding to *C*(*t*_*k*_) and ${\hat{N}}_{A\theta ,j}\;\left(t_{k-1}\right)$, respectively), which explains the use of the notation *p*(*y*_*k*,*j*_|*x*_*k*_, *y*_*k*−1,*j*_). The available information at time step *k* is the set of measurements $Y_{k}=\left\{y_{i}\in R^{N_{s}}:i=1,\ldots ,k\right\}$. Note that other biological effects on the concentration are not considered in the model (16) and (17). The “cell state” as an example of such effects will be included as a hidden Markov state in the later part of this article.

The above model can be extended to a multiple-state (vector x) system where concentrations at different spatial locations are measured by separate sets of CNT sensors, which can be useful in cases where one deals with a spatially distributed concentration profile and/or multiple signal molecules over a large sensing area. In this case, the concentrations and therefore the measured data at different locations can be correlated through the mass transfer phenomena, which can be represented by mass transport models such as diffusion equation [29]. To communicate the essence of the problem in a simple and transparent manner, this article focuses on estimation of concentration at a single location, using single or multiple sensors.

#### Kalman filter

The Bayesian approach offers a systematic way to combine prior knowledge, state and observation models, and measurement information into an informative estimate of the state (i.e., *a posteriori* probability density function (PDF) of the state *p*(*x*_*k*_|*Y*_*k*_)). For linear Gaussian systems, the Kalman filter (KF) enables a recursive construction of the exact PDF of the state estimate, which is parameterized by the mean and covariance. Kalman filtering can be applied to the exact probability distribution model (13) by approximation of the exact PDF by a Gaussian distribution function.

The binomial distribution *B*(*n*, *p*) has the mean of *np* and the variance of *np*(1 − *p*) and can be approximated by a normal distribution with the same mean and variance, N(np,np(1−p))[30]. In this work, the two binomial distributions in the exact observation model can be approximated by

$${\tilde{N}}_{A\theta ,j}\left(t_{k}\right)∼N\left(\mu_{j}^{F},{\left(\sigma_{j}^{F}\right)}^{2}\right)*N\left(\mu_{j}^{E},{\left(\sigma_{j}^{E}\right)}^{2}\right)$$ (18)

$$\mu_{j}^{F}={\hat{N}}_{A\theta ,j}\left(t_{k-1}\right)\lambda^{F}\left(t_{k}\right)$$ (19)

$$\sigma_{j}^{F}={\hat{N}}_{A\theta ,j}\left(t_{k-1}\right)\lambda^{F}\left(t_{k}\right)\left(1-\lambda^{F}\left(t_{k}\right)\right)$$ (20)

$$\mu_{j}^{E}=\left(N_{T}-{\hat{N}}_{A\theta_{j}}\left(t_{k-1}\right)\right)\lambda^{E}\left(t_{k}\right)$$ (21)

$$\sigma_{j}^{E}=\left(N_{T}-{\hat{N}}_{A\theta ,j}\left(t_{k-1}\right)\right)\lambda^{E}\left(t_{k}\right)\left(1-\lambda^{E}\left(t_{k}\right)\right)$$ (22)

Convolution of the two Gaussian distributions $N\left(\mu_{1},\sigma_{1}^{2}\right)$ and $N\left(\mu_{2},\sigma_{2}^{2}\right)$ is a Gaussian distribution with $N\left(\mu_{1}+\mu_{2},\sigma_{1}^{2}+\sigma_{2}^{2}\right)$ [31], so the observation model can be approximated by

$${\tilde{N}}_{A\theta ,j}\left(t_{k}\right)∼N(\mu_{j}^{F}+\mu_{j}^{E},{\left(\sigma_{j}^{F}\right)}^{2}+{\left(\sigma_{j}^{E}\right)}^{2})$$ (23)

Hence, the Gaussian-approximated observation model for the *j*th sensor is defined by

$$y_{k,j}=h\left(x_{k,j},y_{k-1,j}\right)+v_{k,j},\;\forall j=1,\ldots ,N_{s}$$ (24)

where *h*(*x*_*k*,*j*_, *y*_*k*−1,*j*_) is same as the mean in (23) and *v*_*k*,*j*_ is zero-mean Gaussian noise with the variance of (23).

The KF method can be summarized in a recursion of prediction and correction steps, starting from an initial guess defined by the mean ${\hat{x}}_{1|1}$ and the covariance *P*_1|1_. Given the posterior mean and covariance of *x*_*k*_, the mean and covariance of the prior PDF of next state *x*_*k*+1_ is

$${\hat{x}}_{k|k-1}={\bar{A}}_{k-1}{\hat{x}}_{k-1|k-1}$$ (25)

$$P_{k|k-1}={\bar{A}}_{k-1}P_{k-1|k-1}{\bar{A}}_{k-1}^{T}+Q$$ (26)

where *P* is the covariance of the state and *Q* is the covariance of the process noise *w*_*k*_, and ${\bar{A}}_{k-1}$ follows from the linearization

$${\bar{A}}_{k-1}={\left[\frac{\partial f}{\partial x}\right]}_{x={\hat{x}}_{k-1|k-1}}$$ (27)

The mean and covariance of the posterior PDF is

$${\hat{x}}_{k|k}={\hat{x}}_{k|k-1}+L_{k}\left(y_{k}-\left[\begin{array}{c} h\left({\hat{x}}_{k|k-1,1},y_{k-1,1}\right)\\ \vdots \\ h\left({\hat{x}}_{k|k-1,N_{s}}\;,\;y_{k-1,N_{s}}\right) \end{array}\right]\right)$$ (28)

$$L_{k}=P_{k|k-1}{\bar{C}}_{k}^{T}{\left(R_{k}+{\bar{C}}_{k}P_{k|k-1}{\bar{C}}_{k}^{T}\right)}^{-1}$$ (29)

$$P_{k|k}=P_{k|k-1}-L_{k}{\bar{C}}_{k}P_{k|k-1}$$ (30)

where $L_{k}\in R^{N_{s}}$ is the Kalman gain matrix and ${\bar{C}}_{k}\in R^{N_{s}}$ follows from the linearization

$${\left[{\bar{C}}_{k}\right]}_{j}={\left[\frac{\partial h\left(x_{k,j},y_{k-1,j}\right)}{\partial x_{k,j}}\right]}_{x={\hat{x}}_{k|k-1}}$$ (31)

The covariance matrix $R_{k}\in R^{N_{s}\times N_{s}}$ for the measurement noise can be defined by a diagonal matrix

$${\left[R_{k}\right]}_{jj}=r_{k,j}$$ (32)

where *r*_*k*,*j*_ is same with the variance in (23) for the *j*th sensor.

#### Particle filter

To use the non-Gaussian observation model (17) directly, a sampling-based approach known as particle filtering (PF) can be used. PF is based on a discrete weighted approximation of the true posterior PDF with a set of random samples (particles). If the number of samples becomes extremely large, the approximation converges to the true posterior PDF.

The sequential importance sampling (SIS) algorithm is considered as the current standard of PF [26]. The first step of the algorithm is an initialization of *N* particles and their weights, denoted by $\left\{x_{k}^{i},w_{k}^{i};i=1,\ldots ,M\right\}$. In this step, each particle is sampled from the initial PDF $p\left(x_{1}\right)∼N\left({\bar{x}}_{1},Q_{1}\right)$ and the associated weight is initialized to 1/ *M*. After the initialization, the importance sampling step and the weight update step are repeated. In the importance sampling step, $x_{k}^{i}$, *i* = 1,…, *M* are sampled from an importance density *q*(*x*_*k*_|***Y***_*k*_) which is a user-defined choice. The importance density is commonly chosen as the prior PDF,

$$q\left(x_{k}|Y_{k}\right)=p\left(x_{k}|x_{k-1}\right)$$ (33)

In the weight update step, the weight $w_{k}^{i}$ for each *i* = 1,…, *M* is updated with

$$w_{k}^{i}\propto w_{k-1}^{i}\frac{\prod_{j=1}^{N_{s}}p\left(y_{k,j}|x_{k,j}^{i},y_{k-1,j}\right)p\left(x_{k}|x_{k-1}\right)}{q\left(x_{k}|Y_{k}\right)}$$ (34)

If (33) is substituted into (34), the weight update equation is described as

$$w_{k}^{i}\propto w_{k-1}^{i}\prod_{j=1}^{N_{s}}p\left(y_{k,j}|x_{k,j}^{i},y_{k-1,j}\right)$$ (35)

Based on the samples and normalized weights, the posterior PDF can be approximated as

$$p\left(x_{k}|Y_{k-1}\right)=\sum_{i=1}^{M}w_{k}^{i}\delta \left(x_{k}-x_{k}^{i}\right)$$ (36)

where *δ*(⋅) is the Dirac delta function. The estimated value is commonly calculated as a weighted mean,

$${\hat{x}}_{k|k}=\sum_{i=1}^{M}w_{k}^{i}x_{k}^{i}$$ (37)

In addition, a resampling step can be added to mitigate the degeneracy problem [26]. The degeneracy problem refers to the growing number of samples having negligible weights with iterations. The resampling step eliminates the samples with small weights and concentrates the calculation on those samples with large weights whenever a significant degeneracy problem is detected. After generating a new set of $x_{k}^{i}$ for *i* = 1,…, *M* by resampling, the weights are reset to 1/ *M* as in the initialization step. After resampling, the estimated value ${\hat{x}}_{k|k}$ is calculated as a mean of $x_{k}^{i}$ for *i* = 1,…, *M*.

## Results and Discussion

This case study for testing the two approaches is motivated by the problem of detecting tumor cells through NO and H_2_O_2_ signal molecules. NO generated from vascular endothelial NO synthase (eNOS) correlates with stimulation of angiogenesis. This activity is intimately linked with metastasis of tumor cells since their survival and proliferation are highly dependent on adequate supply of O_2_ and nutrients from blood vessels by diffusion [32–34]. Membrane-associated NADPH oxidases are also found in vascular endothelial as well as smooth muscle cells, and generate H_2_O_2_ as an important signal molecule in angiogenesis. Produced H_2_O_2_ can activate signalling pathways to stimulate tumor cell proliferation and migration [35–37]. Knowledge of how concentrations of these signal molecules change as a cell changes its state can help understand their biological roles in tumor cell growth, which in turn can lead to advances in medical treatments.

The estimation of the concentration of signal molecules from a normal cell is examined first, and then the more complex case of a cell transitioning from a normal state to a tumor state is considered.

### Estimating the concentration of signal molecules from a normal vascular endothelial cell

**Fig 2:**
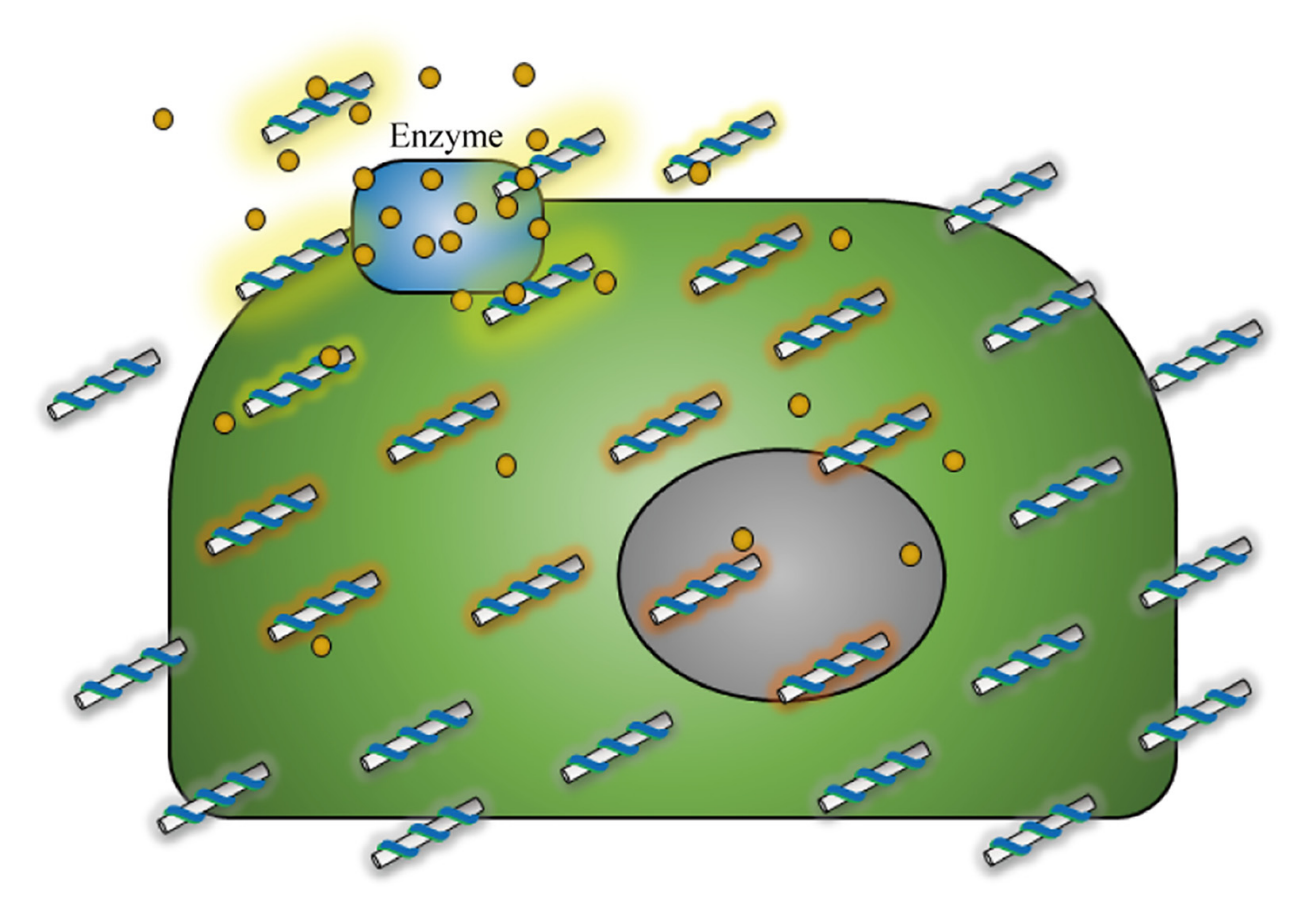
Generation of signal molecules from a membrane-associated enzyme and their detection from a sensor array system on a single cell.

This section develops a state estimation problem for the signal molecules (NO or H_2_O_2_) from vascular endothelial cells. The width and length of the endothelial cells is more than 10 μm [38], which indicates that dozens of SWNT-based sensors can be placed on a single vascular endothelial cell and send multiple stochastic monomolecular adsorption data [7] (Fig 2). Among them, sensors near the enzymes generating signal molecules, where frequent adsorption/desorption events are detected, can be selected and used in the estimation of the local concentration of the signal molecules. The small area proximate to the generator of the signal molecules can be considered as a neighborhood sharing same local concentration that represents the cell state as a whole.

It is difficult to obtain from an experimental setup a large dataset that includes sufficient, representative stochastic variations to render a fair and thorough evaluation of estimation performance. Alternatively, representative stochastic adsorption datasets can be generated from kinetic Monte Carlo (KMC) simulations. Each KMC simulation run can be viewed as a realization of the stochastic system that is described by the CME [39]. The adsorption/desorption process involves fairly simple molecular level events and Zhang et al. 2010 [8] showed that experimental data for this system was well described by the KMC simulation.

In this particular simulation study, the number of adsorbed molecules on the sensor is allowed to range from 0 to 10, so the number of possible discrete states is 11. The length of each run is 2000 s and the sampling time interval is 1 s. The starting state is assumed to be 0 (empty of molecules). Adsorption/desorption parameters, *k*_*A*_ and *k*_*D*_, are chosen as 100 M^−1^s^−1^ and 0.001 s^−1^ respectively, which are taken from [6]. In the normal vascular endothelial cell, the signal molecules are released consistently from the enzyme at a low concentration level (~10 μM) [40]. These dynamics can be simply described as an integrated white noise process,

$$x_{k}=x_{k-1}+w_{k}$$ (38)

**Fig 3:**
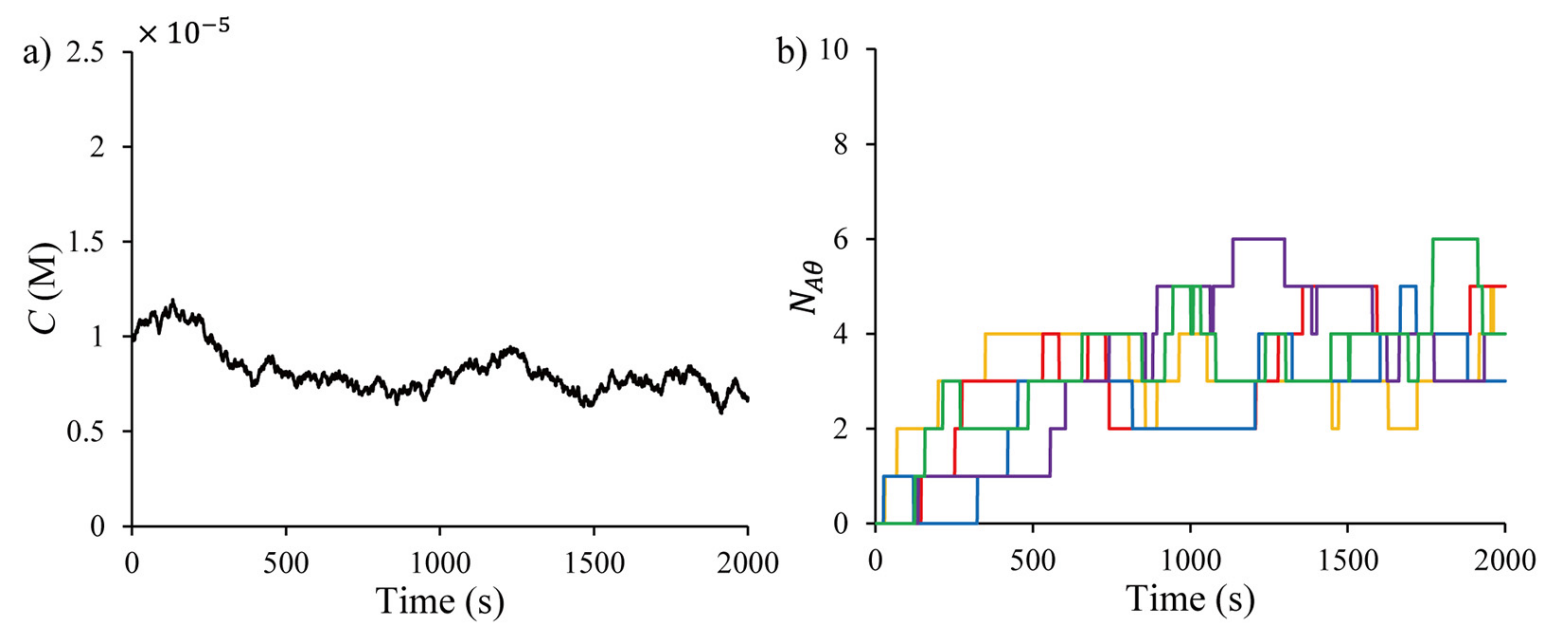
Time plots for (a) the local concentration and (b) associated stochastic adsorption data for a single sensor, generated by KMC simulations.

Fig 3A shows an example time-varying concentration with $w_{k}∼N\left(0,10^{-16}\right)$ in (38) and Fig 3B shows associated five representative realizations of the time profile of the number of adsorbed molecules.

The adsorption data show five distinct time profiles for the same local concentration profile, indicating significant stochastic characteristics of the sensor system at the nano-scale. Other features of the stochastic data shown in Fig 3B are the stepwise (discretized) and reversible variations. In addition, each time profile shows several stationary regions after unpredicted transitions even as the local concentration changes continuously at the bulk phase, which indicates the information-poor characteristic of the dataset, for which accurate modelling is essential for accurate state estimation.

**Fig 4:**
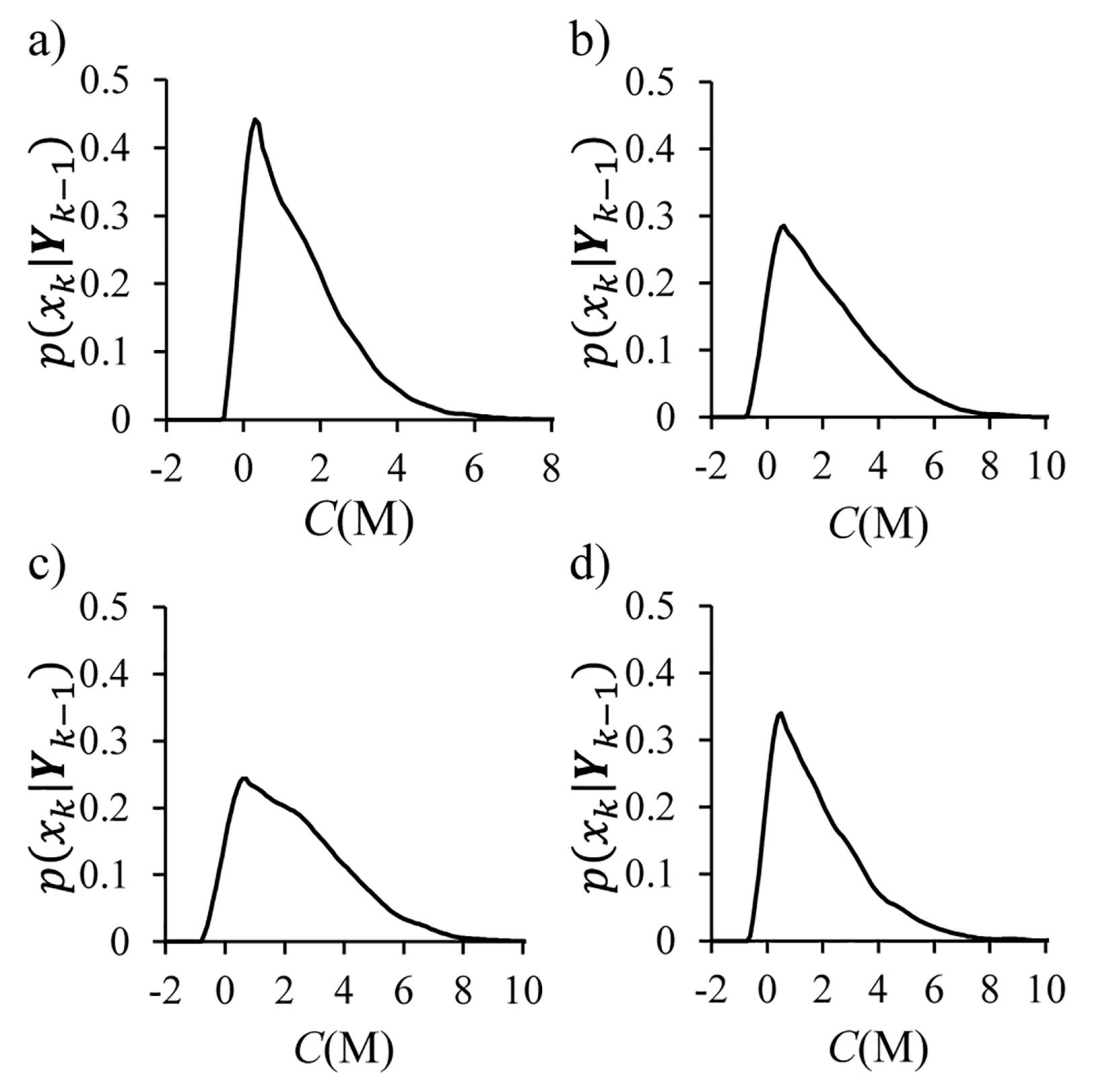
Kernel density reconstruction of the posterior PDFs from the PF at (a) 100 s, (b) 500 s, (c) 1000 s, and (d) 1500 s.

We consider the state estimation problem with increasing number of sensors in the neighborhood measuring the same local concentration. KF and PF (with 200 particles) are compared with increasing number of sensors. KF is based on the Gaussian-approximated observation model, while PF considers the full non-Gaussian stochastic model resulting in the non-Gaussian posterior PDFs as shown in Fig 4. The posterior PDFs are kernel densities reconstructed from the particles of PF for a specific dataset at *t* = 100 s, 500 s, 1000 s, and 1500 s. The overall PDFs show non-Gaussian distributions that are mostly positively skewed.

**Fig 5:**
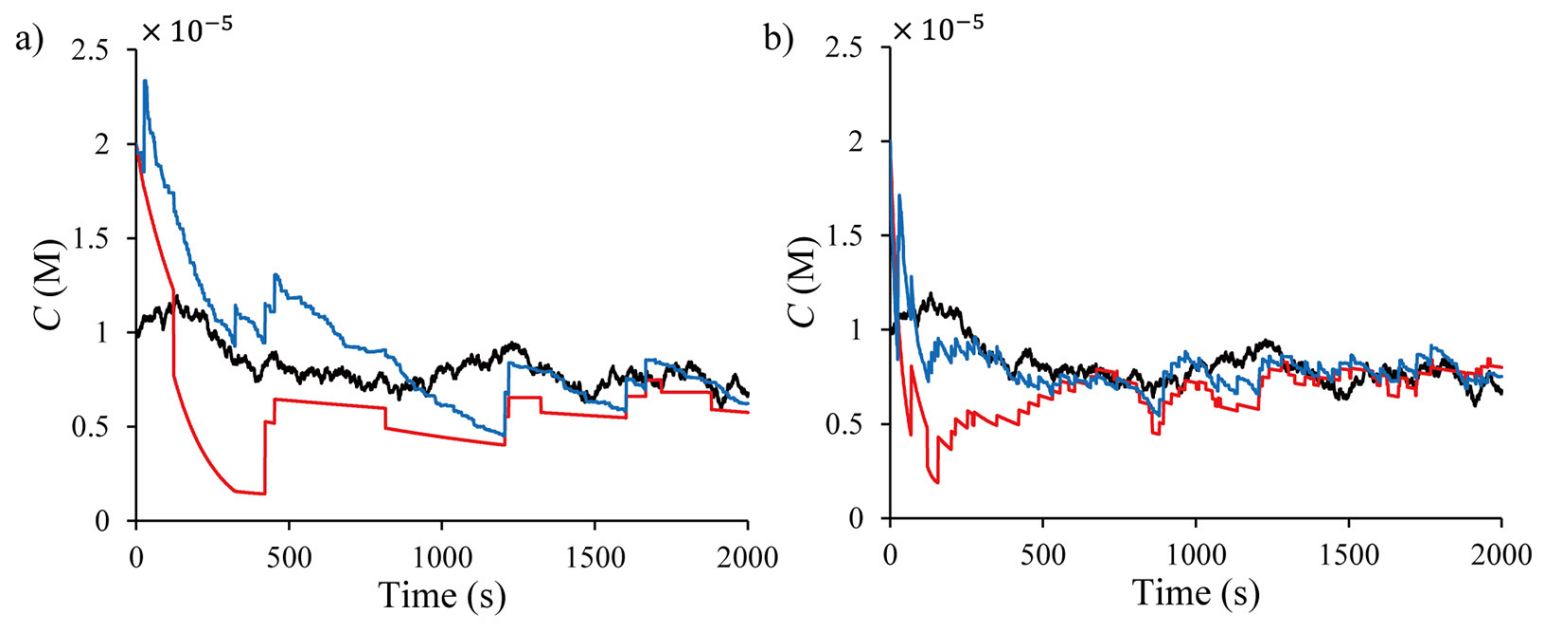
Time plots of the concentration estimates from the KF (red ‒ ∙∙ ‒) and PF (blue ‒ ∙ ‒) for (a) 1 sensor and (b) 5 sensors.

Performance of the two estimation methods can be compared by observing how well the estimates track the true concentration profile throughout the run time from a wrong initial guess (${\bar{x}}_{1}=2\times 10^{-5},Q_{1}=1\times 10^{-5}$). Plots of the estimates for the 1-sensor and 5-sensor cases are shown in Fig 5. For the 1-sensor case, the PF estimates follow the true concentration more closely than the KF (Fig 5A). For the 5-sensor case, the gap between the PF and KF estimates is reduced as long time (Fig 5B).

For quantitative comparison, the root-mean-square-errors (RMSEs) of the estimated concentrations per run are averaged over 100 runs that generated different adsorption/desorption data from different local concentration profiles (2000-sample dataset per one run). The averaged RMSE is defined by

$$\text{RMSE}=\frac{\sum_{i=1}^{N_{R}}\sqrt{\frac{\sum_{k=1}^{N_{T}}{\left(C_{\text{true},i,k}\times 10^{5}-{\hat{C}}_{i,k}\times 10^{5}\right)}^{2}}{N_{S}}}}{N_{R}}$$ (39)

where *N*_*S*_ is the number of samples in one run, *N*_*R*_ is the number of runs, *C*_true,*i*,*k*_ is the true local concentration value, and ${\hat{C}}_{i,k}$ is the estimate for the *k*th sample time of the *i*th run.

In Table 1, for all cases, the RMSEs of the estimates from PF are smaller than from KF. The difference in the RMSEs of the two methods slowly decreases with increasing number of sensors in the neighborhood, while the computation time of PF is higher and increases more rapidly than that of KF. If the objective is only a nominal state estimate, the benefit of rigorous stochastic modelling in the state estimation is reduced when more information is contained in the dataset (through the use of multiple sensors). Of course, a disadvantage of the KF for any number of sensors is that it is not capable of estimating the non-Gaussian character of the distribution of the state estimates.

**Table 1:** Averaged RMSEs of estimates from the PF and KF and associated computation time (in seconds) with increasing number of sensors.

	# of sensors	1	5	10	20
Average RMSE^1^	PF	0.5038	0.3129	0.2457	0.2074
	KF	0.5356	0.3333	0.2521	0.1972
Average computational time^1^ ^,^ ^2^	PF	0.0035	0.0135	0.0275	0.0519
	KF	<0.0001	0.0001	0.0001	0.0001

^1^The values are averaged over 100 runs.

^2^The computation time was recorded in seconds using a workstation with 3.40 GHz CPU and 8GB RAM.

### Estimating the signal molecules from a cell having two states

**Fig 6:**
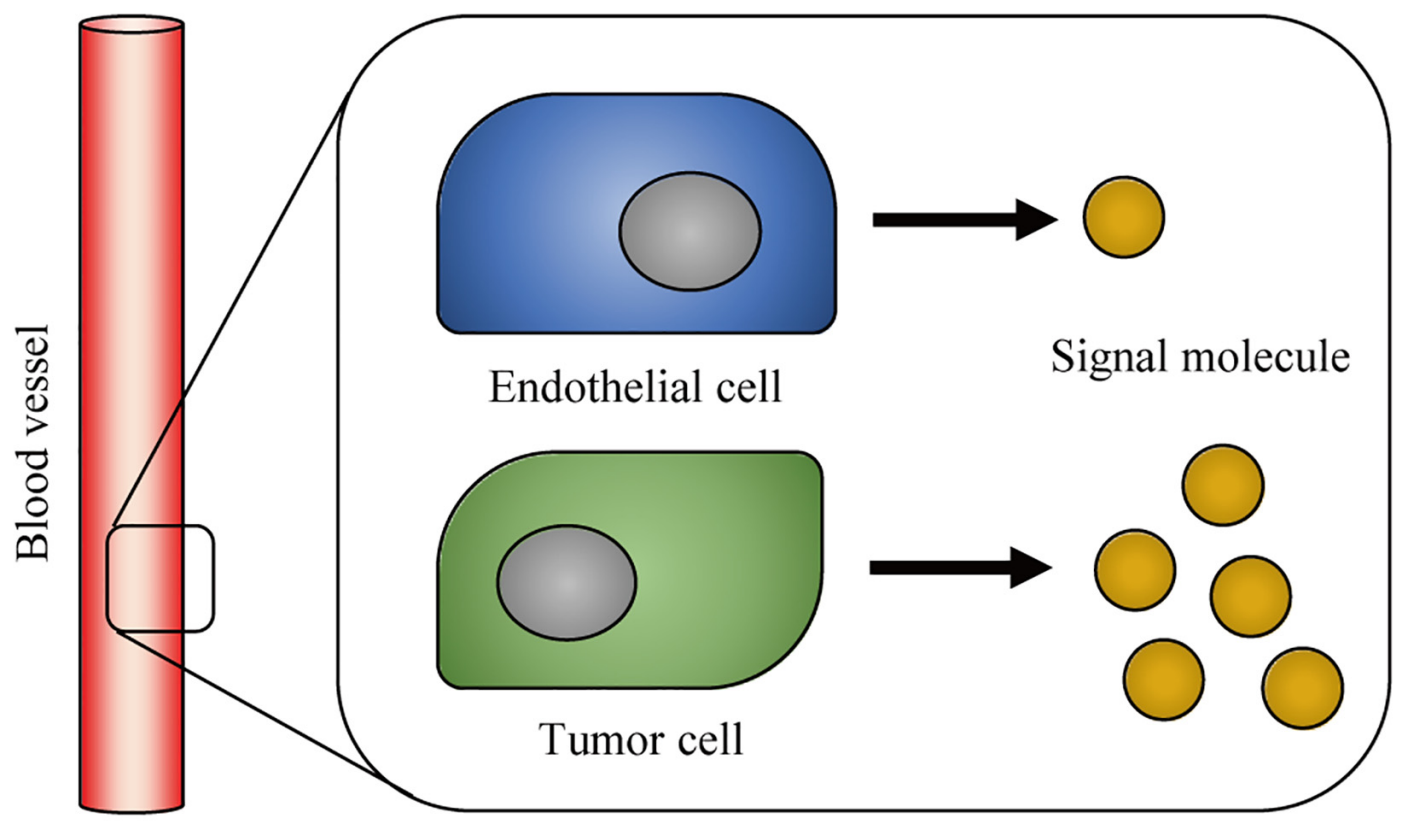
Production of signal molecules from vascular endothelial cells.

In normal vascular endothelial cells, signal molecules generated from the enzymes are at a low concentration level (~10 μM). In tumor vascular endothelial cells, on the other hand, the expression levels and activities of eNOS are abnormally increased compared to the normal endothelial cells (Fig 6), and the elevated level of NO promotes tumor progression and metastasis by inducing angiogenesis as well as tumor cell invasion, proliferation, and migration [41, 42]. For H_2_O_2_, there is also a considerable variation among cells in the concentration level required to initiate a particular biological process. Moreover, it has been observed that different levels of H_2_O_2_ can induce distinct responses within a cell. For example, overproduction of H_2_O_2_ results in proliferation and migration of smooth muscle cells, contributing to atherogenesis and restenosis [43].

#### Hidden Markov model

**Fig 7:**
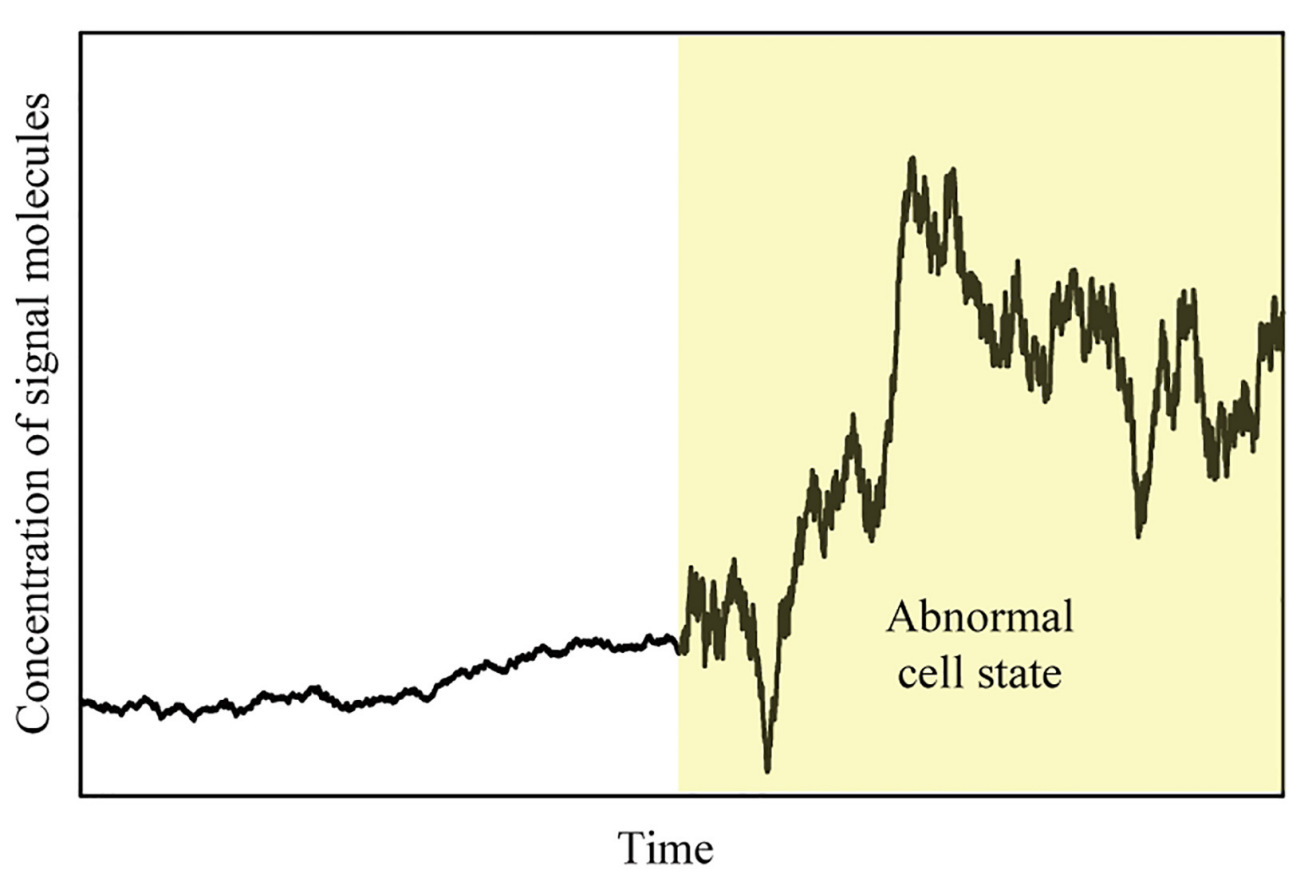
Variation of the concentration of signal molecules with different cell states.

The concentration of the signal molecules affects and is affected by the state of the cell. For real-time state estimation, consideration of all the complex biological processes associated with different cell states is very challenging and linking with concentration variations of the signal molecules can easily become intractable. In addition, the signal molecules are small gaseous molecules showing very fast diffusion (with diffusion coefficients of around 10^−5^ [cm^2^/s]) compared to cell activities in tissues [40]. In this context, we simplify the system to having two states: a normal state and an abnormal state. In the normal state, the signal molecules are released consistently at a low concentration level. In the abnormal state, the concentration of the signal molecules increases (drifts) rapidly to a new elevated level. Fig 7 illustrates a possible concentration variation in the vascular endothelial cell as it transitions to the abnormal state (based on data generated from the artificial Hidden Markov model).

Such a pattern in Fig 7 can be characterized as a mixture of quiescent and drifting phases, which is called “intermittent drift.” A hidden Markov model (HMM) can be used for modelling such shifts in the disturbance pattern [44, 45]. HMM represents a useful class of statistical models where a hidden state, H∈{1,2,…,H} transitions probabilistically among possible states in a Markovian fashion. In this work, each member of the set H represents a particular system state, for example, “normal cell” or “tumor cell” states. Mathematically, a finite-state Markov chain is a sequence of random integers, *r*_*k*_, where the transition probability matrix Π has elements defined by

$$\pi_{ij}=\text{Pr}\left(r_{k}=j|r_{k}=i\right),\;i,j\in H$$ (40)

$$\sum_{j=1}^{H}\pi_{ij}=1$$ (41)

Based on the transition probability matrix, the intermittent drift in the concentration of the signal molecule, *x*_*k*_ can be described by

$$x_{k}=x_{k-1}+w_{r_{k}}\;,\;r_{k}\in 1,\;2$$ (42)

$$\pi_{11}=\pi_{22}\approx 1$$ (43)

$$\pi_{12}=\pi_{21}<1$$ (44)

where “1” indicates the normal cell and “2” indicates the tumor cell. The $w_{r_{k}}$ is a white Gaussian noise with covariance $Q_{r_{k}}$ defined by

$$Q_{r_{k}=1}\approx 0$$ (45)

$$Q_{r_{k}=2}\gg Q_{r_{k}=1}$$ (46)

Since there is only a low probability of switching once the system enters a particular regime, a diagonally dominant Π is employed, as reflected in (43) and (44). Note that the actual regime is usually not known with complete certainty and must be inferred from measurements. Additional behavior could be incorporated into the model by introducing more hidden states (e.g. other transitional cell states or environmental effects on the local concentration) with appropriate accompanying stochastic models for them.

#### Second-order generalized pseudo-Bayesian algorithm

For using KF for a Markov jump system represented by (42), the generalized pseudo-Bayesian estimation algorithm of order 2 (GPB2) has been suggested as an effective sub-optimal filter [46]. Let ${\hat{x}}_{k|k}\left(r_{k-1},r_{k}\right)$ denote the estimate conditioned on the two most recent hidden state realizations. Similarly, the corresponding estimation error covariance is represented as *P*_*k*|*k*_(*r*_*k*−1_, *r*_*k*_). The main idea is to generate multiple Gaussian distributions from KF for all possible trajectories of the last two hidden states, and combine them into a single Gaussian distribution, parameterized by $\left\{{\hat{x}}_{k|k}\;,P_{k|k}\right\}$. A recursive scheme is characterized by two steps: “branching” and “merging.”

Starting with $\left\{{\hat{x}}_{k-1|k-1}\left(r_{k-1}\right),P_{k-1|k-1}\left(r_{k-1}\right)\right\}$, the branching step is to obtain the set $\left\{{\hat{x}}_{k|k}\left(r_{k-1},r_{k}\right),P_{k|k}\left(r_{k-1},r_{k}\right)\right\}$ through the prediction and correction steps of KF. The merging step involves the law of total probability and Bayes’ rule to collapse the products from the branching step as

$${\hat{x}}_{k|k}\left(r_{k}\right)=\sum_{r_{k-1}=1}^{2}{\hat{x}}_{k|k}\left(r_{k-1},r_{k}\right)\;p\left(r_{k-1}|r_{k},Y_{k}\right)$$ (47)

$$P_{k|k}\left(r_{k}\right)=\sum_{r_{k-1}=1}^{2}\left[{\left\{{\hat{x}}_{k|k}\left(r_{k-1},r_{k}\right)-{\hat{x}}_{k|k}\left(r_{k}\right)\right\}}^{2}+P_{k|k}\left(r_{k-1},r_{k}\right)\right]p\left(r_{k-1}|r_{k},Y_{k}\right)$$ (48)

$$p\left(r_{k-1}|r_{k},Y_{k}\right)=\frac{1}{c_{1}}p\left(y_{k}|r_{k-1},r_{k},Y_{k-1}\right)\;p\left(r_{k}|r_{k-1}\right)\;p\left(r_{k-1}|Y_{k-1}\right)$$ (49)

where *c*_1_ is a constant ensuring that *p*(*r*_*k*−1_|*r*_*k*_, ***Y***_*k*_) sums to unity, and *p*(*y*_*k*_|*r*_*k*−1_, *r*_*k*_, ***Y***_*k*−1_) is related to the correction step of KF in the branching step. A point estimate is obtained from

$${\hat{x}}_{k|k}=\sum_{r_{k-1}=1}^{2}{\hat{x}}_{k|k}\left(r_{k}\right)\;p\left(r_{k}|Y_{k}\right)$$ (50)

$$P_{k|k}=\sum_{r_{k-1}=1}^{2}\left[{\left\{{\hat{x}}_{k|k}\left(r_{k}\right)-{\hat{x}}_{k|k}\right\}}^{2}+P_{k|k}\left(r_{k}\right)\right]\;p\left(r_{k}|Y_{k}\right)$$ (51)

$$p\left(r_{k}|Y_{k}\right)=\frac{1}{c_{2}}\sum_{r_{k-1}=1}^{2}p\left(y_{k}|r_{k-1},r_{k},Y_{k-1}\right)\;p\left(r_{k}|r_{k-1}\right)\;p\left(r_{k-1}|Y_{k-1}\right)$$ (52)

where *c*_2_ is a constant ensuring that *p*(*r*_*k*_|***Y***_*k*_) sums to unity.

#### Markov chain Monte Carlo algorithm

Adapting PF to the Markov jump system is relatively simpler than KF. Starting with $\left\{x_{k-1}^{i}\left(r_{k-2},r_{k-1}\right),w_{k-1}^{i}\left(r_{k-2},r_{k-1}\right)\right\}$, samples $x_{k}^{i}\left(r_{k-1},r_{k}\right)$ for *i* = 1,…, *M* are generated from the same importance density of (33) for all possible trajectory for the recent hidden Markov states, *r*_*k*−1_ = 1, 2 and *r*_*k*_ = 1, 2. This approach is called the *Markov chain Monte Carlo* (MCMC) algorithm [47]. The weight update Eq (35) is modified by including, *p*(*r*_*k*_, *r*_*k*−1_|*Y*_*k*_),

$$w_{k}^{i}\left(r_{k-1},r_{k}\right)\propto w_{k-1}^{i}\left(r_{k-1},r_{k}\right)\;p\left(r_{k},r_{k-1}|Y_{k}\right)\prod_{j=1}^{N_{s}}p\left(y_{k,j}|x_{k,j}^{i}\left(r_{k-1},r_{k}\right),y_{k-1,j}\right)$$ (53)

Finally, the point estimate can be obtained by

$${\hat{x}}_{k|k}=\sum_{r_{k-1}=1}^{2}\sum_{r_{k}=1}^{2}\sum_{i=1}^{M}w_{k}^{i}\left(r_{k-1},r_{k}\right)x_{k}^{i}\left(r_{k-1},r_{k}\right)$$ (54)

#### Detection of tumor cell activity

As stated before, two regimes are considered in the system: the normal cell state and the tumor cell state. The objective is to detect a regime change through the local concentration variations of the signal molecules seen from the nano sensors. The reference work [7] investigates the effect of a growth factor, which stimulates cell growth, proliferation, and differentiation on the H_2_O_2_ generation in living cells. From the 3000 s observation after the stimulation with the growth factor at *t* = 0, it was observed that the H_2_O_2_ concentration level increased immediately and reached a maximum in the time range between 600 s and 1800 s. This observation indicates that the tumor cell activity and its effect on the local concentration of signal molecules can be prolonged for a long time (~ 30 min).

**Fig 8:**
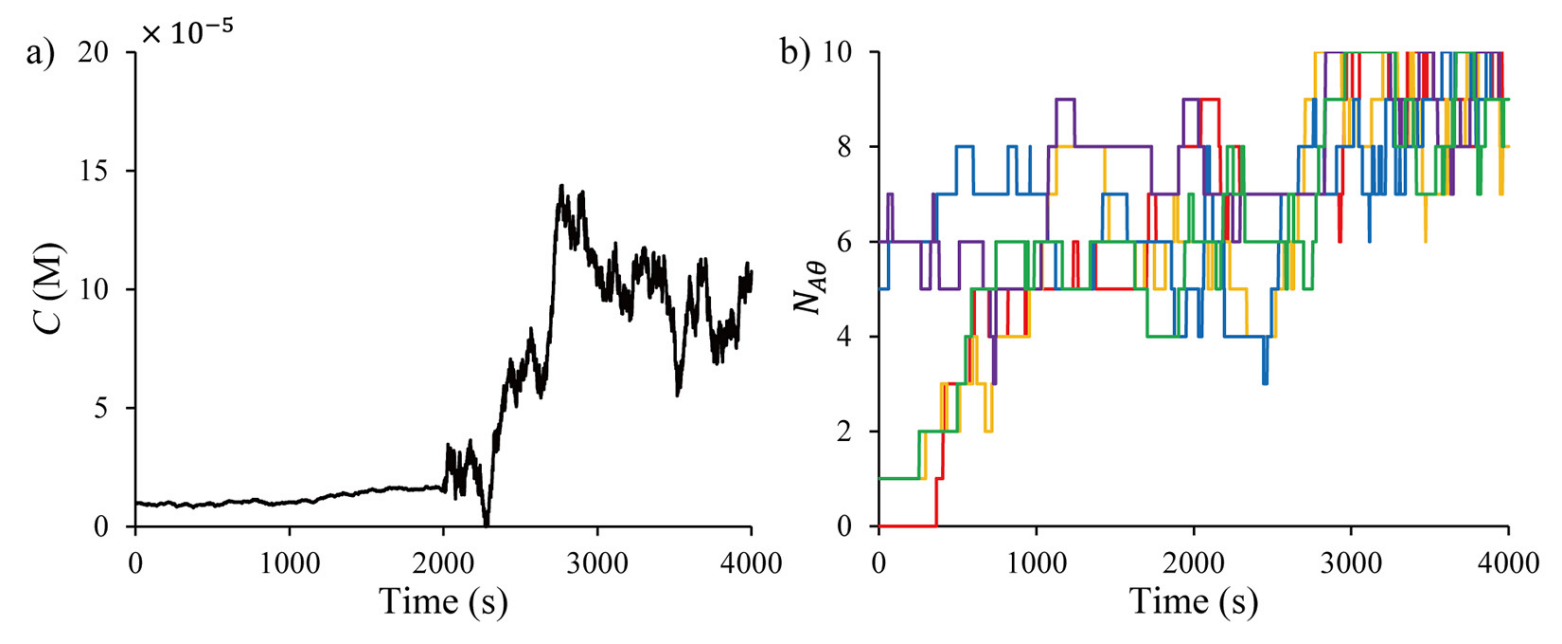
Time plots of (a) the local concentration affected by the tumor cell activity and (b) associated stochastic adsorption data, generated by running KMC simulations.

Based on this data, stochastic adsorption/desorption profiles were generated from KMC simulation. The number of adsorption sites on the sensor is 10 and the length of each run is 4000 s with the sampling time interval of 1 s. The starting state is assumed to be a random integer less than 10 (partly occupied) and the *k*_*A*_ and *k*_*D*_ are assumed to be 100 M^−1^s^−1^ and 0.001 s^−1^. Eq (42) is used in the state estimation as the state model. At the ‘normal cell’ state, the local concentration is stable and affected only by low-level noise ($w_{k}∼N\left(0,10^{-16}\right)$). When the cell becomes a tumor cell, the local concentration becomes elevated by high-level noise ($w_{k}∼N\left(0,5\times 10^{-14}\right)$). The plots in Fig 8 show a representative concentration variation with the tumor cell activity for the time period from 2000 s to 4000 s and five different realizations of the associated time profile of the number of adsorbed molecules.

For this switching system, KF with the GPB2 algorithm (shortly, KF-GPB2) and PF with the MCMC algorithm (shortly, PF-MCMC) are designed and compared. The hidden Markov chain used the state transition probability matrix

$$\Pi =\left[\begin{array}{cc} 0.99 & 0.01\\ 0.01 & 0.99 \end{array}\right]$$ (55)

**Fig 9:**
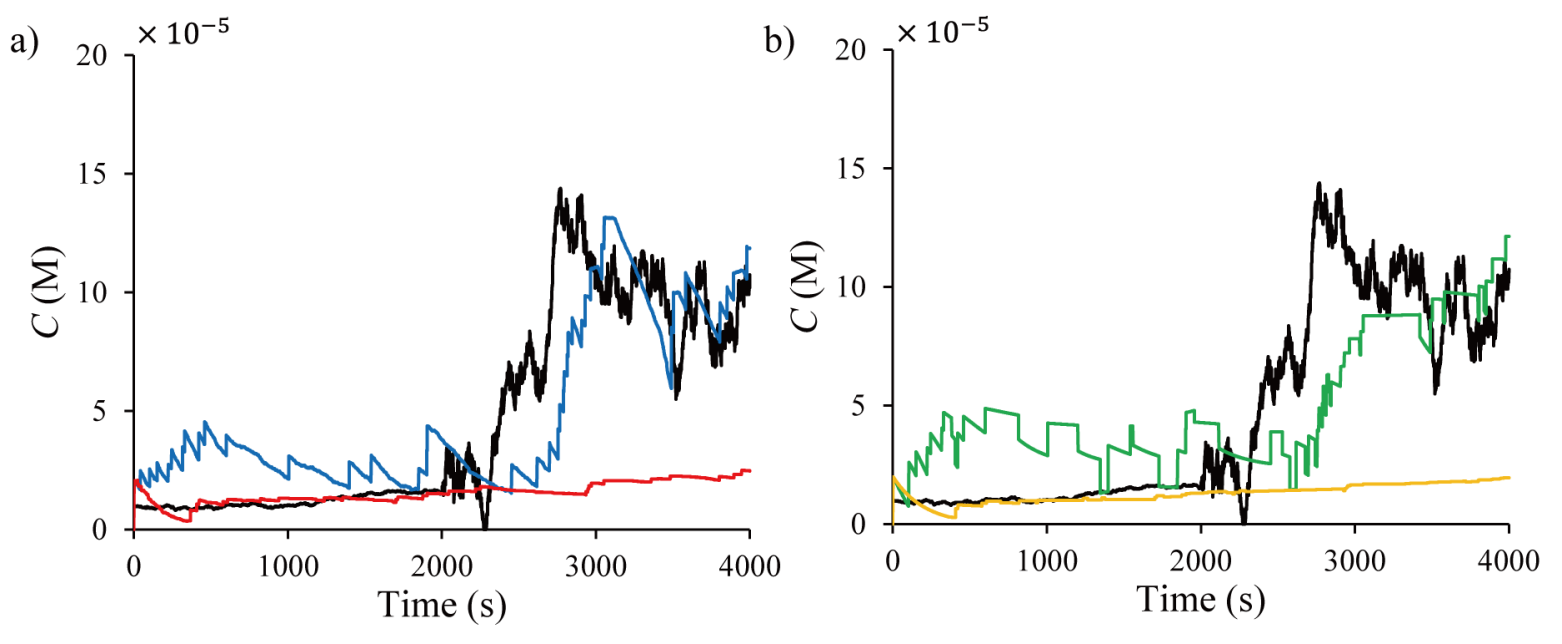
Time plots of the concentration estimates from (a) PF (red ‒ ∙ ‒) and PF-MCMC (blue ‒ ∙∙ ‒) and (b) KF (orange ‒ ∙ ‒) and KF-GPB2 (green ‒ ∙∙ ‒) for one sensor.

Fig 9 shows the state estimates of PF, PF-MCMC, KF, and KF-GPB2 for one sensor. The basic PF and KF used in Section 4 cannot effectively follow the concentration drift caused by the tumor cell activity, while the PF-MCMC and KF-GPB2 estimators follow the drift much better. Among the methods, the estimates from PF-MCMC are closer to the true concentration dynamics and show less fluctuations compared to KF-GPB2.

**Fig 10:**
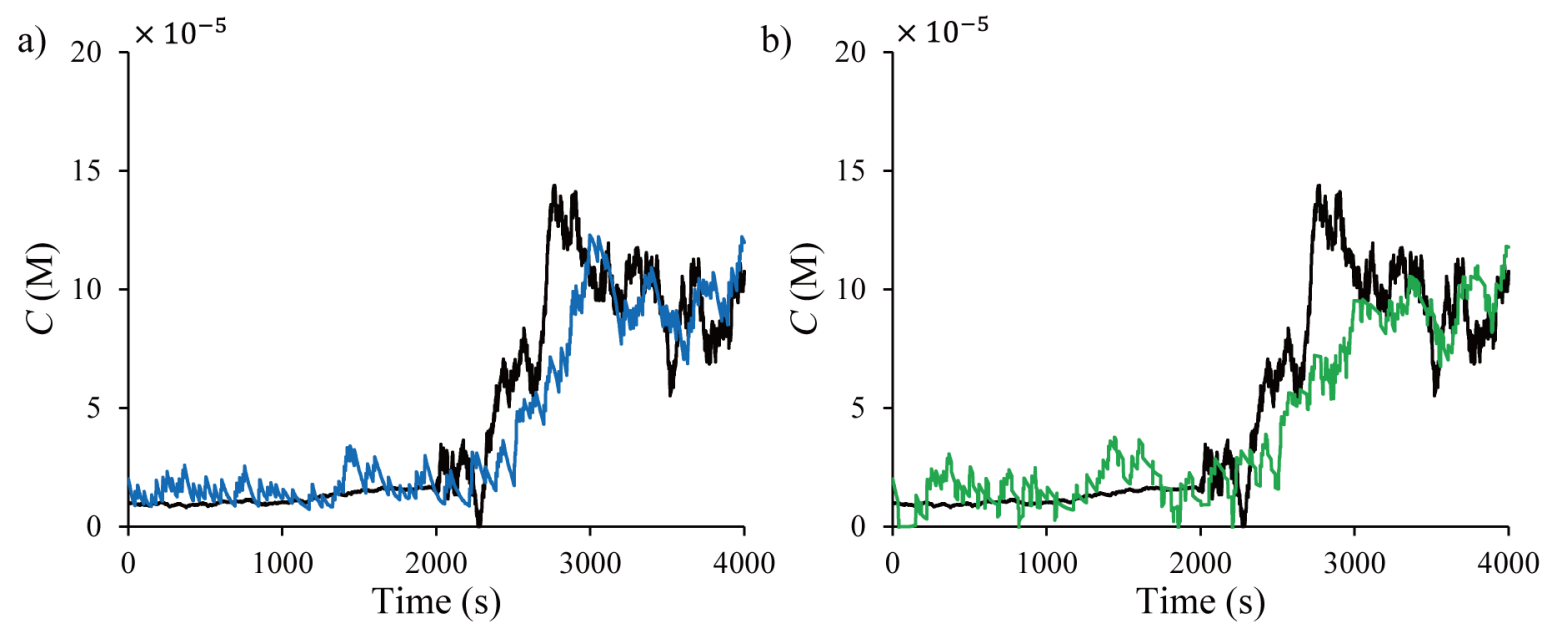
Time plots of the concentration estimates from (a) PF-MCMC (blue ‒ ∙∙ ‒) and (b) KF-GPB2 (green ‒ ∙ ‒) for five sensors.

In both PF-MCMC and KF-GPB2, the state estimates are improved further when more stochastic adsorption/desorption data are available, obtained from multiple sensors, as shown in Fig 10.

Table 2 shows the averaged RMSEs and computational time for PF-MCMC and KF-GPB2 based on 100 runs that generated different adsorption/desorption data from different tumor cell activities. In both methods, the RMSEs of the estimates decrease with increasing number of sensors in the neighborhood with PF-MCMC having smaller RMSE values than KF-GPB2. Though the computation time of PF-MCMC is larger than KF-GPB2, it is far less than the sampling time of 1 s.

**Table 2:** Averaged RMSEs and of the estimates from PF-MCMC and KF-GPB2 and associated computation time for increasing number of sensors.

	# of sensors	1	5	10	20
Average RMSE^1^	PF-MCMC	2.3748	1.9180	1.7544	1.6190
	KF-GPB2	2.6095	2.0816	1.8984	1.7542
Average computational time^1^ ^,^ ^2^	PF-MCMC	0.0120	0.0474	0.0839	0.1637
	KF-GPB2	0.0016	0.0076	0.0126	0.0250

^1^The values are averaged over 100 runs.

^2^The computation time was recorded in seconds using a workstation with 3.40 GHz CPU, 8GB RAM.

In a real application, the neighborhood region proximate to the enzyme should be very small considering the short-life time and high diffusivity of the signal molecules. Therefore, less than 5 sensors might be valid in the state estimation for a single cell [7]. In this context, PF-MCMC can be recommended if accurate estimates of the local concentration of the signal molecules are needed with such limited information.

## Conclusions

Two stochastic state estimation methods–Kalman filtering (KF) and particle filtering (PF)–were investigated for estimating the time-varying local concentration of signal molecules from stochastic monomolecular adsorption/desorption data on the surface of the carbon-nanotube (CNT)-based sensors. In addition, the second-order generalized pseudo Bayesian estimation (GPB2) algorithm and the Markov chain Monte Carlo (MCMC) algorithm were incorporated into KF and PF respectively, for detecting latent drift in the concentration affected by different states of a cell. The stochastic nature of the adsorption data from each CNT-based sensor was fully modelled by using the chemical master equation (CME). In addition, intermittent concentration variations of the signal molecules were modelled by a hidden Markov model. Performances of the state estimators with the sensor array system were compared through a case study employing KMC simulation. The PF-MCMC combination showed the highest accuracy while having reasonable computation time.

Use of drugs affecting the production of signal molecules by inhibiting the associated enzyme or directly scavenging the signal molecules appears to be a promising strategy to inhibit angiogenesis and therefore tumor growth [48, 49]. In order to control the modification of signal molecules in a precise manner, further understanding of various factors involved such as the timing, concentration, and location is required. The proposed state estimators have promise in this endeavor.

## Supporting Information

S1 Dataset Original data including true concentration profiles, stochastic adsorption profiles and concentration estimates for plotting Figs 3, 5, 8, 9 and 10. (XLSX)
